# *De novo* genome assessment of *Limosilactobacillus reuteri C4*, a potential probiotic lactic acid bacterium isolated from porcine Ileum

**DOI:** 10.1128/mra.00823-24

**Published:** 2025-05-30

**Authors:** Dikonketso Shirleymay Matjuda, Tshifhiwa Paris Mamphogoro

**Affiliations:** 1 Gastro-Intestinal Microbiology and Biotechnology Unit, Agricultural Research Council-Animal Production, Irene, Pretoria, South Africa 623417 , Irene, Gauteng, South Africa; University of Maryland School of Medicine, Baltimore, Maryland, USA

**Keywords:** probiotics, genomes, *Limosilactobacillus reuteri*, porcine, ileum

## Abstract

*Limosilactobacillus reuteri* is a lactic acid bacterium with several probiotic properties. Here, we present the draft genome sequence of *L. reuteri* isolated from the porcine ileum with a genome size of 2.1 Mb. The average genome sequence had a G + C content of 43.71% with 113 contigs, 54 RNA, and 2,012 protein-coding sequences.

## ANNOUNCEMENT

*Limosilactobacillus reuteri* is an indigenous inhabitant of the animal gut known for its probiotic effects on the host (1). Probiotics are live microorganisms that are currently a functional health product of great interest in humans and animals worldwide. *Limosilactobacillus reuteri* is an indigenous bacterium in the gastrointestinal tract (GIT) and has been reported to naturally settle among various vertebrates, including humans, pigs, rodents, and chickens (1–3); therefore, it generally has good adhesion and adaptability in the GIT. Therefore, bioinformatics analysis of whole-genome sequencing data is a more convenient and comprehensive method to explore the potential of probiotic strains at the gene level.

In this study, strain C4 was isolated from the porcine ileum at the Agricultural Research Council-Animal Production, Irene, South Africa (25°53′59.6″S, 28°12′51.6″E). The isolation method was adopted from reference 4. Briefly, bacterial cells were obtained by diluting 1 mL of each porcine ileum content sample into 9 mL of sterile 0.85% saline solution using the serial dilution method. The organism was cultured on de Man, Rogosa, and Sharpe (MRS) solid agar plates supplemented with 0.05 g/L cysteine-HCl and incubated anaerobically using an Anaerobic Gas Pack (Oxoid) for 48 h at 37°C. The genomic DNA was extracted from overnight liquid culture using a Quick-DNA Miniprep Kit (Zymo Research, Irvine, CA, USA) following the manufacturer’s instructions, and the genomic library was prepared using the NEBNext Ultra II FS DNA Library Prep Kit (New England Biolabs, Ipswich, MA, USA). The libraries were paired-end sequenced (2 × 150 bp) on the Illumina MiSeq using v3 chemistry (Illumina, USA) at the Biotechnology Platform, Agricultural Research Council, Onderstepoort, South Africa, producing 4,937,570 paired-end reads.

The paired-end reads were quality evaluated using FastQC version 0.11.9 (5), and low-quality reads were filtered using Trimmomatic version 0.36 (6) on the Kbase platform (6), allowing the removal of low-quality reads <Phred30. The trimmed reads were assembled using SPAdes version 3.13.0 (7). Contamination (3.78%) and completeness (96.22%) of the genome were assessed using CheckM (version 1.2.2) (8). Statistics of genome assembly were generated using QUAST (9) and revealed the total size of the genome assembly to be about 2.1 Mb (GC content, 43.71%; *N*_50_, 35,167 bp, *L*_50_, 37, and 813.65× coverage). Gene annotation was performed using Prokaryotic Genome Annotation Pipeline version 6.6 (10), which revealed 113 contigs with 2,122 genes (total), 2,012 coding genes, 54 RNA, 51 tRNAs, 3 ncRNAs, and 56 total pseudogenes.Default parameters were used for all software. AntiSMASH v6.0.0 (11) was used to analyse secondary metabolites. This technique identified genes that are linked to probiotic activities including; acid tolererance, bile salt tolerance, adhesion to epithelial cells, and antioxidant production. The draft genome information of *Limosilactobacillus reuteri C4* reported here provides the basis for understanding the properties that make C4 a probiotic strain with potential applications as a component of animal gut health.

## Data Availability

The WGS shotgun was deposited in GenBank under accession number JAXOJV000000000. The draft genome sequence was deposited on the NCBI website under the SRA accession number SRR28800653, BioProject accession number PRJNA1050914, and BioSample accession number SAMN38757438.

## AFTER EPUB

[This article was published on 30 May 2025 with an error in the Acknowledgments. The error was corrected in the current version, posted on 4 June 2025.]
